# Matrix metalloproteinases 2 and 9 (gelatinases A and B) expression in malignant mesothelioma and benign pleura

**DOI:** 10.1038/sj.bjc.6600920

**Published:** 2003-05-13

**Authors:** J G Edwards, J McLaren, J L Jones, D A Waller, K J O'Byrne

**Affiliations:** 1 University Department of Medical Oncology, Osborne Building, Leicester Royal Infirmary, Leicester LE1 5WW, UK; 2 Department of Respiratory Medicine and Thoracic Surgery, Glenfield Hospital NHS Trust, Groby Road, Leicester LE3 9QP, UK; 3 Department of Obstetrics and Gynaecology, University of Leicester, Robert Kilpatrick Building, Leicester Royal Infirmary, Leicester LE1 5WW, UK; 4 Department of Pathology, University of Leicester, Robert Kilpatrick Building, Leicester Royal Infirmary, Leicester LE1 5WW, UK

**Keywords:** malignant mesothelioma, matrix metalloproteinases, prognosis

## Abstract

Matrix metalloproteinases (MMPs), in particular the gelatinases (MMP-2 and -9), play a significant role in tumour invasion and angiogenesis. The expression and activities of MMPs have not been characterised in malignant mesothelioma (MM) tumour samples. In a prospective study, gelatinase activity was evaluated in homogenised supernatants of snap frozen MM (*n*=35), inflamed pleura (IP, *n*=12) and uninflammed pleura (UP, *n*=14) tissue specimens by semiquantitative gelatin zymography. Matrix metalloproteinases were correlated with clinicopathological factors and with survival using Kaplan–Meier and Cox proportional hazard models. In MM, pro- and active MMP-2 levels were significantly greater than for MMP-9 (*P*=0.006, *P*<0.001). Active MMP-2 was significantly greater in MM than in UP (*P*=0.04). MMP-2 activity was equivalent between IP and MM, but both pro- and active MMP-9 activities were greater in IP (*P*=0.02, *P*=0.009). While there were trends towards poor survival with increasing total and pro-MMP-2 activity (*P*=0.08) in univariate analysis, they were both independent poor prognostic factors in multivariate analysis in conjunction with weight loss (pro-MMP-2 *P*=0.03, total MMP-2 *P*=0.04). Total and pro-MMP-2 also contributed to the Cancer and Leukemia Group B prognostic groups. MMP-9 activities were not prognostic. Matrix metalloproteinases, and in particular MMP-2, the most abundant gelatinase, may play an important role in MM tumour growth and metastasis. Agents that reduce MMP synthesis and/or activity may have a role to play in the management of MM.

Malignant mesothelioma (MM) is an aggressive and invariably fatal serosal tumour of increasing incidence usually associated with asbestos exposure. Median survival in the UK is less than 6 months from the date of diagnosis (Edwards *et al*, 2000). Malignant mesothelioma is characterised by rapid progression and invasion of intrathoracic structures. Although commonly perceived as a tumour that rarely metastasises, nodal disease is present in about 40% of patients undergoing extrapleural pneumonectomy (Sugarbaker and Norberto, 1998), while distant metastases are seen in at least 70% at the time of death (Rusch *et al*, 1991). Malignant mesothelioma responds poorly to conventional modes of therapy including surgery, chemotherapy and radiotherapy (Sterman *et al*, 1999). Staging by the conventional criteria of Tumour–Nodes–Metastasis (TNM) is difficult in MM: that proposed by the International Mesothelioma Interest Group (IMIG) is the most recent TNM staging system (Rusch, 1995). However, in a large series of patients managed by radical surgery and chemoradiotherapy, this staging system failed to stratify survival (Sugarbaker *et al*, 1999). For MM, prognostic scoring systems incorporating clinicopathological variables have been proposed by the European Organisation for Research and Treatment of Cancer (EORTC) (Curran *et al*, 1998) and the Cancer and Leukemia Group B (CALGB) (Herndon *et al*, 1998). These systems have been useful in predicting survival in our patient population (Edwards *et al*, 2000).

Novel prognostic factors, such as microvessel density (MVD), a marker of the intensity of angiogenesis, have been examined in MM. High MVD is associated with a poor outcome in this disease (Kumar-Singh *et al*, 1997; Edwards *et al*, 2001), in keeping with the results from similar studies in other solid tumours (Dickinson *et al*, 1994; Fox *et al*, 1994; Cox *et al*, 2000c). Furthermore, in other solid tumours, such as nonsmall cell lung cancer, novel biological staging systems are being developed, which contribute independently to TNM stage (Cox *et al*, 2000a; O'Byrne *et al*, 2001).

Proteolysis of the extracellular matrix and basement membrane, by proteases such as matrix metalloproteinases (MMPs), is a central part of tumour growth and metastasis. The stromal remodelling mediated by these enzymes also facilitates tumour angiogenesis. MMPs are a family of zinc-dependent enzymes, which are implicated in the growth of primary and secondary tumours (Cox *et al*, 1999). Overexpression of MMPs, in particular gelatinase A (MMP-2), gelatinase B (MMP-9) and stromelysin-3 (MMP-11), is related to tumour progression and metastasis in solid tumours including gastric (Schwartz, 1996), colon (Karakiulakis *et al*, 1997) and lung cancer (Cox *et al*, 2000b).

Reports using semiquantitative gelatin zymography have correlated MMP-2 and/or MMP-9 activity with survival and disease progression in several solid tumour types (Brown *et al*, 1993b; Tokuraku *et al*, 1995; Ikebe *et al*, 1999; Papathoma *et al*, 2000; Lengyel *et al*, 2001; Di Nezza *et al*, 2002; Waas *et al*, 2002). The use of SDS in the buffers activates latent gelatinase isoforms, which enables assessment of both the latent (pro) and active bands of enzymatic activity. Although MMP expression has been described in MM cell lines (Harvey *et al*, 2000; Liu *et al*, 2001), the activity of MMPs has not been described in MM tumour samples. We have assessed, by gelatin zymography, the activities of MMP-2 and -9 in specimens of benign pleura and MM.

## MATERIALS AND METHODS

### Patients and samples

Patients referred to the regional Department of Cardiothoracic Surgery for surgical management of pleural diseases were identified prospectively. Patients were due to undergo surgery for biopsy, management of pleural effusion, empyema or pneumothorax or for palliative or radical surgery for malignant mesothelioma. Clinicopathological prognostic factors, including CALGB (Herndon *et al*, 1998) and EORTC (Curran *et al*, 1998) prognostic groups were recorded before surgery, as described previously (Edwards *et al*, 2000). For the patients undergoing radical surgery, the IMIG TNM pathological stage was derived.

Samples of MM, inflamed pleura (IP) or uninflammed, benign pleura (UP) were collected at surgery from consecutive patients. Surgery in MM patients (*n*=35, 23 epithelioid, seven mixed cellularity, five sarcomatoid) was performed either for diagnostic biopsy or for symptom control. IP patients (*n*=12) had empyema thoracis and underwent decortication of visceral pleura and/or parietal pleurectomy. UP specimens were taken from patients (*n*=15) at the time of pleurectomy for primary spontaneous pneumothorax. Samples were assessed macroscopically and representative areas (e.g. tumour nodules) were processed as formalin-fixed, paraffin-embedded blocks for diagnostic evaluation, or snap frozen in liquid nitrogen and stored at −80°C for zymography. Standard diagnostic immunohistochemistry was performed to confirm the diagnosis, using antibodies including CEA, BerEP4, AUA-1 and HBME1, where appropriate. Following surgery, the detailed histopathological report and diagnostic slides were reviewed by a second pathologist to confirm the diagnosis.

### Gelatin zymography

Samples were homogenised mechanically at 4°C in a zymography homogenisation buffer (containing the protease inhibitor phenylmethylsulphonyl fluoride (PMSF, Sigma-Aldrich, Poole, UK, at 0.1 mM), centrifuged and the supernatant refrozen. Protein concentrations were derived with a Bradford assay. Wells of a gelatin preimpregnated SDS–polyacrylamide gel (Novex, Frankfurt, Germany) were loaded with samples (3.75 *μ*g protein) and run for 100 min at 125 mV in Tris-glycine SDS running buffer (Novex Frankfurt, Germany), during which time the bromophenol blue front reached the bottom of the gel. Positive controls included the Type I Collagenases AG770 (Chemicon International, Harrow, UK) and C-0130 (Sigma-Aldrich); the molecular weight marker SeeBlue Pre-Stained Standards (Novex) was also used. Gels were renatured in zymogram renaturing buffer (Novex) for 45 min and incubated overnight at 37°C in zymogram developing buffer (Novex). After staining with 0.5% Coomassie Blue G-250, destaining with 30% methanol, 10% acetic acid solution revealed clear bands of gelatinolytic activity. Within each assay, all samples were assessed simultaneously using the same reagents and batch of gels. Gels were digitally photographed and band densitometry assessed using a computer image analysis system (Scion Image, Frederick, Maryland, USA), to give a semiquantitative assay of enzymatic activity. Densitometry of each band was assessed blind to the diagnostic group three times and the mean score used for statistical analysis. The semiquantitative nature of this protocol was validated by densitometric analysis of serial dilutions of the two samples with the strongest bands. Assays were repeated for all samples and the results obtained for different runs were correlated. Three samples were run in each gel as internal positive controls. All gels were run under identical conditions with the same batch of reagents and densitometry results standardised between gels.

### Statistics

Gelatin zymography was performed and results analysed prospectively. Statistical analysis was performed using the SPSS software system (SPSS for Windows Version 9.0, SPSS Inc., Chicago, USA). Differences in densitometry readings between groups were assessed with the Mann–Whitney *U* and Kruskal–Wallis tests, where appropriate. Cancer-specific survival curves were estimated using the Kaplan–Meier method and the log-rank test was used to assess the statistical significance of differences between groups. A Cox proportional hazards regression model (Cox, 1972) was used to identify the impact of prognostic factors on survival and estimate hazard ratios and 95% confidence intervals (CIs). The assumption of proportional hazards was assessed graphically by plotting log[−log(survivor)] against log(time) for each group. Prognostic variables identified by univariate analysis, with *P*<0.1, were analysed in multivariate Cox models. A forward, stepwise selection procedure was used, with variables being added to the model according to a partial likelihood ratio test, using an entry criterion of *P*<0.05.

## RESULTS

### Patients

Overall median survival in the MM group was 7.5 months. Six months and 1 year survival rates were 66 and 34% respectively. The definitive surgical procedures performed were radical surgery in 14 patients (11 extrapleural pneumonectomies and three radical decortications), palliative debulking surgery (Martin-Ucar *et al*, 2001) in 16 and biopsy alone in five patients. Although patients who underwent radical surgery survived longer than those who did not (*P*=0.04, log rank), the surgical procedure performed was not an independent factor in subsequent multivariate analyses.

Three patients (8.6%) died within 30 days of surgical biopsy. Two patients developed postoperative respiratory failure and died on days 11 and 23, while a third suffered a cardiac arrest following an acute intrathoracic haemorrhage on day 10. These three patients were excluded from subsequent survival analyses.

### Gelatin zymography

#### Methods validation

Serial dilution of the two strongest MM samples (between 1 and 30 *μ*g) demonstrated that at protein loads of greater than 10 *μ*g per well, the densitometry values reached a plateau (data not shown). The relation of protein load to densitometry value was linear up to and including 10 *μ*g protein per well, which was that used in the main experiments. When samples were run on different days and the correlation between them assessed, correlation coefficients of 0.936, 0.863, 0.973 and 0.946 were obtained for pro-, active MMP-9, pro- and active MMP-2 bands, respectively (*P*<0.0001). The variation between gels of the control samples was minimal (data not shown).

### Gelatin zymography results

Initially, 22 MM samples were compared with the UP and IP specimens. Densitometry readings for each group and Mann–Whitney *P* values are shown in Table 1

**Table 1:** Zymogram densitometry values in MM and benign pleura

	***n***	**Pro-MMP-9**	**Active MMP-9**	**Pro-MMP-2**	**Active MMP-2**
MM	22	1996	1400	2743	1936
		(1268–2724)	(953–1848)	(2102–3385)	(1183–2689)
UP	15	1934	1423	2297	1038
		(1466–2403)	(1063–1783)	(1543–3051)	(648–1428)
IP	14	3642	2380	3485	2499
		(2324–4960)	(1591–3170)	(2132–4837)	(1005–3994)
					
Mann–Whitney *P* values:					
MM *vs* UP		0.5	0.6	0.4	0.04
IP *vs* MM		0.02	0.009	0.7	0.7
IP *vs* UP		0.05	0.01	0.1	0.07

Data are displayed as mean arbitrary densitometry units (95% CI). MM=malignant mesothelioma; UP=uninflammed pleura; IP=inflammed pleura; MMP-9=gelatinase B; MMP-2=gelatinase A.

. Subsequently, the gelatin zymography was repeated in a larger series of 35 MM samples alone. The latter group was used to examine correlations with clinicopathological factors and survival.

**Figure 1:**
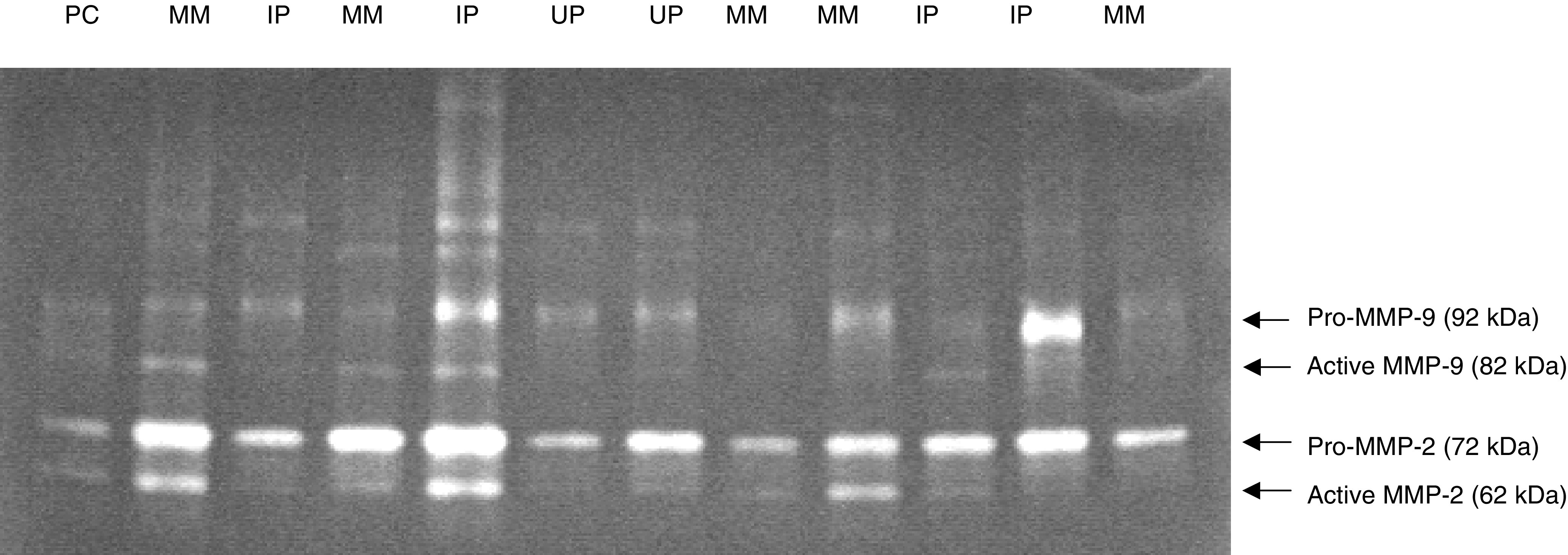
Gelatin zymogram of malignant mesothelioma (MM), inflamed pleura (IP) and uninflamed pleura (UP). Snap-frozen samples were homogenised and the supernatant proteins resolved in a gelatin preimpregnated polyacrylamide gel. Gels were developed to show clear bands of gelatinolysis, correlating with the latent, pro- and active isoforms of MMP-2 and -9.

Gelatinolytic bands corresponding to pro-MMP-9 and pro- and active MMP-2 were seen in all 35 cases of MM. No detectable active MMP-9 was seen in nine of the 35 MM cases (26%). MMP-2 was the predominant gelatinase seen in MM (Figure 1

). Among the 35 MM cases, pro-MMP-2 and -9 levels were mean 2600 (95% CI 2130–3070) and 1640 (960–2330) respectively (*P*=0.006). Values were 1540 (1300–1770) and 850 (580–1110) for the active isoforms (*P*<0.001). It was possible to determine densitometry values for all gelatinase isoforms in each of the UP and IP cases (Table 1).

### Correlation with clinicopathological variables

**Figure 2:**
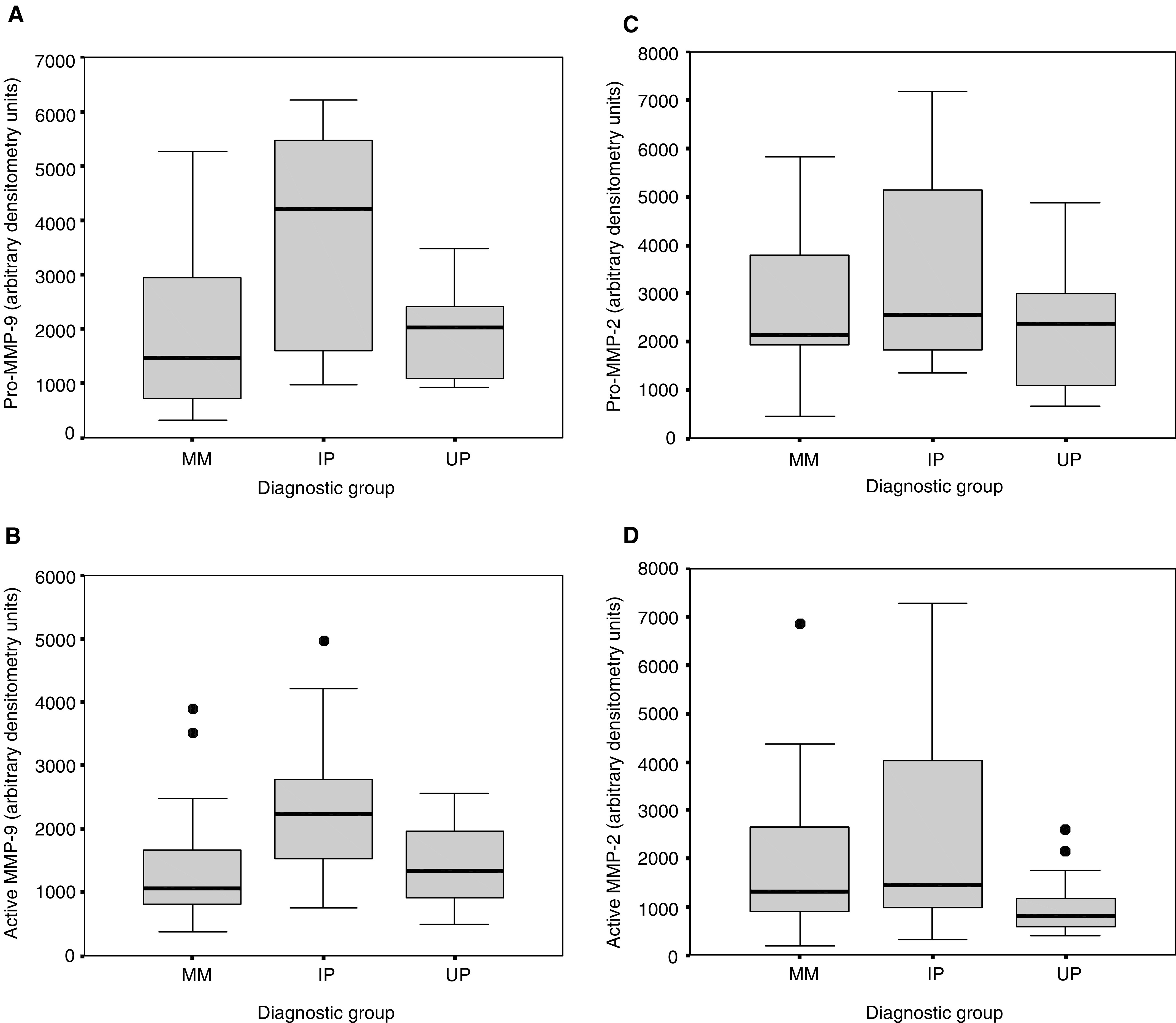
Boxplots showing the differences in MMP activity between MM (*n*=22), UP (*n*=12) and IP (*n*=15). Data are shown for pro-MMP-9 (**A**), active MMP-9 (**B**), pro-MMP-2 (**C**) and active MMP-2 (**D**). Horizontal bars represent the median values, boxes the interquartile range, vertical bars the 10–90% range and solid circles the outliers. MMP enzyme activity was measured with semiquantitative gelatin zymography and is expressed by arbitrary densitometry units per milligram protein.

Active MMP-2 was significantly elevated in MM compared with UP specimens (*P*=0.04, Table 1, Figure 2

). There were no differences between MM and UP groups with regard to MMP-9 activity. There was significantly greater pro- and active MMP-9 gelatinolytic activity in IP specimens compared to the UP (*P*=0.05, 0.01 respectively) and MM (*P*=0.02, 0.009 respectively) groups. There was no significant difference in pro- and active MMP-2 gelatinolytic activity between IP and either MM or UP specimens.

Preoperative white cell count was associated with greater total MMP-9 (sum of pro- and active MMP-9, *P*=0.0006) and a trend towards a similar result was seen with thrombocytosis (*P*=0.06). There were no significant correlations between gelatinases and gender, age, presence of chest pain or weight loss, cell type or performance status. There was no correlation between any MMP isoform and either the EORTC or CALGB prognostic scores. Pathological staging was performed only for the 13 patients who underwent radical surgery. Nodal stage was N0 in seven, N1 in one and N2 in five patients. Pro-, active and total MMP-2 activities were greater in those with extrapleural nodal metastasis than without (Table 2

**Table 2:** Correlation between MMP-2 and MMP-9 activities and clinicopathological variables

**Variable**		***n***	**Pro-MMP-9**	**Active MMP-9**	**Total MMP-9**	**Pro-MMP-2**	**Active MMP-2**	**Total MMP-2**
Cell type	Epithelioid	23	1176	608	1693	2104	1199	3418
	Nonepithelioid	12	816	633	1419	2422	1491	4087
	*P*		0.082	0.344	0.237	0.281	0.889	0.314
								
White blood cells	<8.3 × 10^9^ l^−1^	13	788	511	1279	1988	1193	3426
	⩾8.3 × 10^9^ l^−1^	22	1167	638	1796	2468	1559	3895
	*P*		0.065	0.109	0.006	0.585	0.824	0.539
								
Platelets	<400 × 10^9^ l^−1^	22	824	582	1419	2279	1449	3547
	⩾400 × 10^9^ l^−1^	13	1176	608	1917	2105	1199	3771
	*P*		0.172	0.757	0.060	0.864	0.670	0.811
								
Haemoglobin	<14 g dl^−1^	22	824	629	1412	2279	1491	3814
	⩾14 g dl^−1^	13	1367	502	1764	1955	1193	3012
	*P*		0.037	0.158	0.142	0.517	0.597	0.375
								
CALGB prognostic group	Groups 1, 2	10	1247	562	1567	2218	1090	3265
	Groups 3,4	17	820	662	1693	2241	1622	3485
	Groups 5,6	8	1036	608	1353	2446	1113	4087
	*P*		0.598	0.363	0.999	0.890	0.187	0.592
								
EORTC prognostic group	Low risk	20	895	611	1448	2279	1440	3547
	High risk	15	1157	608	1764	2092	1199	4018
	*P*		0.463	0.534	0.217	0.714	0.841	0.790
								
IMIG nodal status	N0, N1	8	1091	189	1353	1093	1086	3069
	N2	5	1042	662	1727	3643	1852	6097
	*P*		0.884	0.075	0.107	0.019	0.013	0.008
								
IMIG stage	II	2	962	383	1345	1566	1317	2883
	III	11	1043	625	1483	2847	1725	4677
	IV	8	1000	409	1437	1923	1184	3207
	*P*		0.840	0.689	0.755	0.173	0.797	0.353

The median densitometry value for each isoform is quoted. The Mann–Whitney *U*-test was used for the comparison of two groups and the Kruskal–Wallis Test for greater than two groups. Total MMP activity equals the sum of pro- and active enzyme values. MMP-9=gelatinase B; MMP-2=gelatinase A; CALGB=Cancer and Leukemia Group B; EORTC=European Organisation for Research and Treatment of Cancer; IMIG=International Mesothelioma Interest Group.

). There were no associations seen between gelatinases and pathological T or overall IMIG TNM stages.

### Correlation with survival

**Figure 3:**
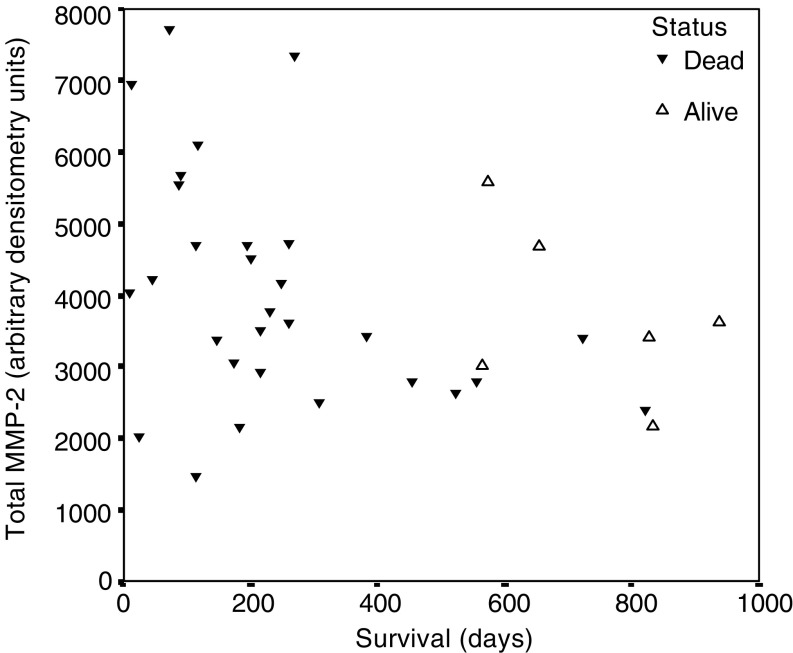
Scatterplot showing the relation between survival and total MMP-2 activity (sum of pro-MMP-2 and active MMP-2 band arbitrary densitometry units). There was a trend towards worse survival in patients with increasing values (*P*=0.08, Cox proportional hazards).

In univariate Cox proportional hazards analysis, trends towards increasing pro- and total MMP-2 activities (as continuous variables) and poor prognosis were seen (*P*=0.08, HR 1.0003, 95% CI 1.0000–1.0006 and *P*=0.08, HR 1.0002, 95% CI 1.0000–1.0005 respectively, Figure 3

). However, although trends towards poor prognosis were seen with the categorical analyses, these did not reach statistical significance in either univariate Cox proportional hazards models (*P*=0.16 and 0.19 respectively) or the log-rank test (*P*=0.15 and 0.19). There was no significant relation between poor prognosis and active MMP-2, or total, pro-, or active MMP-9. Similarly, the ratio of pro- to active MMP-2 or -9 (activation ratio) and total gelatinolytic activity (sum of all four isoforms) were not prognostic.

### Multivariate analyses

In addition to the association between the operation performed and survival (as outlined above), clinicopathological factors significant in univariate survival analysis were weight loss >5% (*P*=0.0006), nonepithelioid cell type (*P*=0.0049) and preoperative haemoglobin <14 g dl^−1^ (*P*=0.03). These were entered with either pro-MMP-2 or total MMP-2 gelatinolytic activity into Cox proportional hazards multivariate models. Both pro-MMP-2 (*P*=0.03) and total MMP-2 (*P*=0.04) were independent prognostic factors in conjunction with weight loss (*P*=0.0008), whereas the other factors entered were not. Similarly, high pro-MMP-2 (*P*=0.04) and total MMP-2 (*P*=0.04) contributed independently to the CALGB prognostic scoring system (*P*=0.005 and 0.02 respectively). The EORTC risk groups were not prognostic in univariate analysis in this series (*P*=0.16, log rank) and therefore were not tested in multivariate analysis.

## DISCUSSION

Malignant mesothelioma is characterised by extensive local growth and invasion of intrathoracic organs. This pattern of tumour development suggests an important role for proteases, including the MMPs, in the evolution of the disease. This study demonstrates the consistent expression of active and latent forms of MMP-9 and particularly MMP-2 in MM tumour samples by gelatin zymography. We used whole tumour extracts rather than microdissected extracts since recent evidence has suggested that stromal expression of MMPs is important in tumour progression (Himelstein *et al*, 1994; Nawrocki *et al*, 1997; Polette and Birembaut, 1998; DeClerck, 2000; Kikuchi *et al*, 2000). Gelatin zymography was found to be reproducible, with a low variation between samples run on different days and between the control samples run in each gel. The serial dilution experiment confirmed the semiquantitative nature of the assay when 10 *μ*g protein was loaded to each well.

The benign UP control group specimens were obtained from patients undergoing pleurectomy for primary spontaneous pneumothorax. However, even this group may not display true baseline expression of MMPs. Both acute and chronic inflammatory changes were noted in the histopathology reports of the diagnostic tissue blocks from the ‘UP’ specimens that accompanied the snap-frozen samples used in this study. It is possible to speculate, therefore, that we have underestimated the degree of upregulation of MMPs in MM, and indeed in IP, compared to UP.

The patterns of gelatinolytic activity seen in MM, IP and UP are likely to reflect both the nature and extent of the inflammatory cell infiltrate in these conditions, and between benign and malignant mesothelial cells. The predominant gelatinase expressed by macrophages and neutrophils in inflammatory conditions such as emphysema, asthma, sepsis and inflammatory bowel disease is MMP-9 (Finlay *et al*, 1997; Mautino *et al*, 1997; Pugin *et al*, 1999), which is in keeping with the significantly elevated activity of this enzyme seen in IP specimens compared to either MM or UP samples. Furthermore, the observation that total MMP-9 was significantly greater in MM patients with a WCC greater than 8.3 × 10^9^ l^−1^ supports this contention. The levels of active MMP-2 were found in this study to be significantly higher in MM than UP. This finding suggests that the MMP-2 activity seen in MM may be more specific than MMP-9 to the carcinogenic process.

Activities of MMP-2 and -9 isoforms have been investigated by gelatin zymography in other solid tumours. Upregulation of MMP-2 is seen in solid tumours including hepatocellular (Maatta *et al*, 2000), colorectal (Liabakk *et al*, 1996; Baker *et al*, 2000; Waas *et al*, 2002) and ovarian cancers (Lengyel *et al*, 2001). With regard to the relation of MMP activity, as assessed by gelatin zymography, with prognosis, results differ between studies and tumour types. In oral squamous cell carcinoma, activities of MMP-2 and -9 correlate with disease-free survival (Yorioka *et al*, 2002). In colorectal cancer, the relations between MMP activities and tumour progression remain unclear. Waas *et al* (2002) found no association between MMP activity and nodal metastasis, but did note significantly lower tumour extract MMP-2 activity in patients with distant metastasis. Baker found that total MMP-2 and -9 levels in colorectal tumour samples, as assessed by gelatinase activity assays, correlated positively with Dukes' stages (Baker and Leaper, 2002). Liabakk, however, demonstrated higher levels of MMP-9 in Dukes' stages A and C (Liabakk *et al*, 1996). Brown found that active MMP-2 correlated with stage in NSCLC (Brown *et al*, 1993b) but not breast cancer (Brown *et al*, 1993a). Pro-MMP-9, but neither MMP-2 isoform, was associated with poor survival in both univariate and multivariate analyses in ovarian cancer (Lengyel *et al*, 2001). No relations were seen in bladder cancer (Papathoma *et al*, 2000), hepatocellular carcinoma (Maatta *et al*, 2000) or soft-tissue sarcomas (Maguire *et al*, 2000).

The numbers in our study are relatively small, particularly with respect to those with an accurate pathological TNM stage. The accuracy of radiological TNM staging in MM remains unclear and therefore we made no attempt to assess the stage of patients who did not undergo radical surgery and detailed pathological assessment. Nonetheless, the positive correlation between MMP-2 activity and nodal status noted here is in keeping with a number of studies in other tumours cited above. Similarly, although our multivariate survival analyses may lack high statistical rigour, our results are provocative and justify confirmatory studies in a larger series of patients.

The regulation of MMP expression (Jones and Walker, 1997) in MM remains to be characterised. Nonetheless, many growth factors known to induce MMP expression are detectable in MM. These include basic fibroblast growth factor (bFGF) (Kumar-Singh *et al*, 1999), hepatocyte growth factor/scatter factor (Tolnay *et al*, 1998), vascular endothelial growth factor (VEGF) (Ohta *et al*, 1999), insulin-like growth factor (IGF)-1, tumour necrosis factor (TNF), transforming growth factors-*α* and -*β* (Bielefeldt-Ohmann *et al*, 1996) and interleukin-8 (Antony *et al*, 1996). The inter-relation between these growth factors and MMP expression in MM tumour samples needs to be investigated. Furthermore, gelatin zymography does not discriminate between free MMPs and those complexed with TIMPs, mRNA for which have been identified in MM cells *in vitro* (Liu *et al*, 2001). Further study is required to address the balance of MMPs with respect to TIMPs in MM tumour samples.

The detection of active gelatinase isoforms in MM samples, particularly MMP-2, is important and suggests that MMP inhibitors may be therapeutic in this disease. Evidence to support this contention comes from both experimental and clinical studies. Inhibition of MMPs reduces tumour growth, invasion and angiogenesis *in vivo* (Maekawa *et al*, 1999). Initial phase III trials of MMP inhibitors in other solid tumours alone or in combination with cytotoxic agents have overall been disappointing (Bissett *et al*, 2002; Bramhall *et al*, 2002a,2002b). Nonetheless, inhibition of MMP activity, with synthetic MMP inhibitors (Drummond *et al*, 1999; Steward, 1999) or with biological agents that downregulate the synthesis and activation of the enzymes such as the selective COX-2 inhibitors (Reich and Martin, 1996; Attiga *et al*, 2000; Pan *et al*, 2001; Abiru *et al*, 2002), deserves clinical investigation as a novel approach to the management of MM.
